# Analysis of Risk Factors Related to Early Implant Failures in Patients Attending a Private Practice Setting: A Retrospective Study

**DOI:** 10.3390/jcm14186546

**Published:** 2025-09-17

**Authors:** Renzo Guarnieri, Rodolfo Reda, Dario Di Nardo, Gabriele Miccoli, Alessio Zanza, Luca Testarelli

**Affiliations:** 1 Private Periodontal Implant Practice, 31100 Treviso, Italy; renzoguarnieri@gmail.com (R.G.); luca.testarelli@uniroma1.it (L.T.); 2 Dentistry Departement, Fondazione Policlinico Universitario Campus Bio-Medico, Via Alvaro del Portillo, 200, 00128 Rome, Italy; rodolforeda17@gmail.com (R.R.); alessio.zanza@uniroma1.it (A.Z.); 3 Department of Oral and Maxillo Facial Sciences, Sapienza University of Rome, 00161 Rome, Italy; miccoligabriele@gmail.com

**Keywords:** risk factors, dental implant abutments design, peri-implantitis

## Abstract

**Background/Objectives:** The aim of the study was to investigate the risk factors related to early implant failures in patients treated in a private implant clinic. **Methods:** The study was retrospectively conducted on 392 patients (mean age = 51.2 ± 15.4 years, 48.9% female and 51.1% male) who received 930 dental implants within the period from 2000 to 2020. Included patients had received at least one implant. Patients were excluded in case of incomplete dental records that did not contain the necessary information, including personal information (patient’s name, age, gender, history and current condition of systemic diseases) and treatment protocol record. No patients were excluded on the basis of systemic disease if it did not contraindicate dental implant surgery. Patient-related variables (age, gender, smoking, history of periodontitis, accompanying disease), surgical-related variables (surgical technique, bone augmentation, lateral sinus lift, internal sinus lift, immediate implant placement after tooth extraction, immediate mucosal grafting, insertion torque,) and implant-related variables (implant design, implant level, implant surface, site of implant placement, implants diameter, length and implants brand name) were recorded. **Results:** GEE analysis results showed that the implant-based failure rate before or at the abutment connection stage was 5.8%. Seven factors were identified associated with early implant failures: male gender, smoking, history of radiotherapy and chemotherapy, maxilla implant placement, non-submerged healing method, implant design and implant brand. **Conclusions:** In light of the results obtained, both patient-related variables (gender, smoking, history of radiotherapy and chemotherapy) and variables related to the type of implant, its position and the surgical technique used (maxilla implant placement, non-submerged healing method, implant design and implant brands) were found to be statistically correlated with early failures in this study.

## 1. Introduction

Supported by the scientific basis of “osseointegration” established during the 1970s [1], dental implantology today represents a reliable therapy for the treatment of partial and total edentulism [2]. However, in spite of the predictable clinical outcomes, dental implant failures that require implant removal may occur [3]. Traditionally, according to the time they happen, implant failures can be classified as “early” if they occur before the application of functional loading and “late” if they occur after applying occlusal loading or after the first removal of the provisional restoration in cases of immediate implant loading [4]. Early failures are the result of the inability to achieve osseointegration, while late failures are associated with the inability to maintain osseointegration over time [4]. Early implant failures (at the implant level) have been reported at rates ranging from 0% to almost 6%, while late implant failures range with greater sensitivity, from less than 1% to almost 10%, being influenced by the differences in longer observation periods [4].

Clinically, early-failed implants show mobility, but only half of them show signs of inflammation at the implant site [5,6,7], and the presence of pain is reported in only a few cases [5]. The formation of a fibrotic scar encapsulation has been proposed as an explanation for mobile implants free of any symptoms [8].

Studies that have been conducted on early dental implant failures have identified various potential risk factors, such as smoking habit, history of periodontitis, metabolic disorder, anatomical structure (quality and quantity of bone), surgery technique, use of grafted bone, piezosurgery or conventional drilling, insertion torque, etc., but it is still difficult to identify a clear etiological pathway [5,6,7,8,9,10,11,12,13,14,15,16,17,18,19,20,21,22,23]. Most of these studies have been conducted prospectively in university clinics, where convenient sampling tends to exclude individuals with systemic diseases and other hypothetical risk factors. Therefore, they reported data in a favorable patient population, without considering other components that might negatively impact implant success. Few references have been otherwise reported concerning early implant failures in private clinics. The present study was retrospectively conducted on patients that had been treated in a private practice setting, regardless of their accompanying medical or other conditions, including risk factors which have not been mentioned in previous studies. It aimed to assess the influence of different patient-, surgical-, and implant-related factors on the incidence of the early implant failures in patients treated in private practice settings, regardless of their accompanying medical or other conditions.

## 2. Materials and Methods

This study was retrospectively conducted on patients who experienced implant treatment in a private dental clinic within the period from 2000 to 2020. The study was conducted in accordance with the Declaration of Helsinki and approved by the Ethics Committee of Università Campus Bio-Medico di Roma (protocol code Prot. PAR 30.21 (OSS) ComEt CBM-30/03/2021). The inclusion criteria were patients who had received at least 1 implant. Patients with incomplete dental records that did not contain the necessary information were excluded. The sample size was calculated using G*Power software version 3.1.9.7, where the alpha level of significance was 0.05, the effect size was 0.15 (medium), and the power was 0.9 [24]. With these parameters, a total sample size of at least 900 implants and 300 patients was estimated to have good statistical power to carry out hypothesis testing for this study. A total of 392 patients who received 930 dental implants were included.

Included patients had received at least 1 implant. Patients were excluded in case of incomplete dental records that did not contain the necessary information, including personal information (patient’s name, age, gender, history and current condition of systemic diseases) and treatment protocol record. No patients were excluded on the basis of systemic disease if it did not contraindicate dental implant surgery.

Data Collection. Patient data were extracted from medical, surgical and radiographic records, ensuring accurate categorization and coding for statistical analysis. Two calibrated operators were involved in data collection, with an inter-examiner agreement of >90%. Each record was meticulously reviewed to maintain consistency and reliability.

Patient-related variables included age, gender, smoking, history of periodontitis and accompanying disease (Table 1).

Surgical-related variables included surgical technique, bone augmentation, lateral sinus lift, internal sinus lift, immediate implant placement after tooth extraction, immediate mucosal grafting and insertion torque. (Table 2).

All bone augmentation procedures, including lateral sinus lift and internal sinus lift, were performed with porcine-derived bone (MinerOss, BioHorizons, Birmingham, AL, USA) and porcine-derived collagen membrane (Mem-Lok Pliable^®^, BioHorizons, Birmingham, AL, USA)

Implant-related variables included the site of implant placement, implant diameter, implant length, implant design, implant level and implant brand name (Table 3).

In addition, the dates of implant placement and failed implant removal surgery were also recorded.

Early failures were diagnosed if there was a

(1) Presence of persistent subjective complaints, such as pain, foreign body sensation, and/or dysesthesia;
(2) Presence of recurrent peri-implant infection with suppuration;
(3) Presence of mobility;
(4) Presence of any continuous peri-implant radiolucency.

Statistical Analysis: Statistical analysis evaluations were performed with SPSS 19.0 (SPSS Inc., Chicago, IL, USA). Patient characteristics are presented as frequencies for categorical outcomes. A differentiation between early implant failure and risk factors was performed. Individual associations between implants and early implant failure were calculated using a binary generalized estimating equation model (GEE), adjusted for confounding factors and effects caused by repeated observations. Odds ratios (ORs), which were calculated for the significant factors, were obtained. ORs were considered measures of outcome and exposure. A significance level was set at *p* = 0.05 to determine significant differences in the analysis.

## 3. Results

The patients’ mean age was 51.2 ± 15.4 years; 125 (31.8%) were ≤40 years old, 427 (47.9%) were 41–60 years old and 176 (19.7%) were >60 years old. Female and male patients made up 48.9% and 51.1%, respectively. In total, 53 (13.5%) patients were smokers, 87 patients (22.2%) presented systemic diseases and 358 (91.8%) patients had been subjected to a standard implant placement, while 20 (5.1%) and 12 (3%), respectively, received implants using a lateral sinus lift or internal sinus lift (Table 2). Regarding implants, 325 (34.9%) were placed using one-stage surgery and 605 (65.1%) using two-stage surgery. A total of 307 (33%) implants were associated with a bone augmentation procedure, and 233 (24.7%) were associated with a barrier membrane placement. Additionally, 111 (12%) implants were immediately placed in post-extraction sockets and 93 (10%) were associated with an immediate mucosal graft; 717 (77%) implants were placed in posterior areas [291 (31.3%) in maxilla and 426 (45.8%) in mandible]. A total of 125 (13.5%) implants had a cylindrical design and 805 (86.5%) a conical design; 316 (33.9%) implants were <3.75 mm in diameter (narrow) and 278 (29.8%) were <10 mm in length (short); and 623 (66.9%) implants presented an insertion torque of 25–35 Ncm. (Table 2 and Table 3). The results showed that the implant-based failure rate before or at the abutment connection stage was 5.8. GEE analysis showed a statistically significant correlation of early implant failures with seven factors: male gender, smoking, history of radiotherapy and chemotherapy, maxilla implant placement, non-submerged healing method, implant design and implant brand (Table 4).

## 4. Discussion

The present retrospective observational study was conducted on 930 implants in 392 patients who underwent implant surgery in a private dental clinic, and used a GEE model to identify risk factors for early implant failures. Results showed that gender, smoking, history of radiotherapy and chemotherapy, maxilla implant placement, non-submerged healing method, implant design, and implant brand were statistically correlated with early failures. Most of the published studies on early implant failures have been conducted in university clinics and, for ethical reasons, they reported data in a favorable patient population, without considering other components that might negatively impact implant success. The main strength of the current study is the inclusion of all patients treated within the period from 2000 to 2020, regardless of their accompanying medical or other conditions, considering the relationship between early implant failures and several factors which have not been mentioned in previous studies. The objective is to provide information that might help dentists properly manage risk factors in the oral clinic. Among all the factors taken into consideration, the study identified seven factors associated with early implant failure: male gender, smoking, history of radiotherapy and chemotherapy, maxilla implant placement, non-submerged healing method, implant design and implant brand. The risk of implant failures among men was found to be 1.54 times higher than for women. Whether early implant failures can be influenced by gender is still controversial. Some studies report that gender has an effect on early implant failures [21,25], while others reported a non-significant influence [24,26]. Presumably, our results can be explained by the higher percentage of smokers present among men. In the current study smoking presented high odds ratios in the multivariate analysis. These outcomes agree with studies by Baqain et al. [12] and Olmedo-Gaya et al. [21], who reported a 2- to 3-fold proportion of early implant failures among smokers compared with nonsmokers. It is known that nicotine can induce oxidative stress in peri-implant tissue, affecting the process of osseointegration and increasing the possibility of early implant failure. However, even regarding smoking, the data present in the literature are contradictory, with some studies having reported no statistical correlation between smoking and early implant failures [10,15]. Regarding systemic diseases, the outcomes of the current study showed a significant correlation between a history of radiotherapy and chemotherapy and early implant loss rate. Both ionizing radiation and chemotherapy disrupt host defense mechanisms and hematopoiesis and may interfere with the osseointegration process [27]. Radiation therapy causes osteoclastic and non-osteoclastic resorption. Moreover, tissue damage does not regress at the end of the therapy, causing, even after 6 months, the presence of fibrosis and reduced vascularization [27]. Antitumor drugs can also negatively influence the initial healing process of bone by inducing rapid granulocytopenia, followed by thrombocytopenia [27]. Therefore, there is a need to consider patients’ histories of radiotherapy and chemotherapy before dental implant treatment. Few data are reported in the literature on the relationship between hyperglycemia and early implant failures. In the present study, no significant correlation was found between early implant failures and type I or II diabetic status. Contradictory outcomes are reported in the literature on the relationship between hyperglycemia and early implant failures. This could be justified by an imprecise distinction between the state of the disease (compensated and non-compensated) or by the lack of knowledge of the glycemic values reported by the patients. Most of the investigated diabetic patients in the present study had a well-compensated state of the disease. Therefore, our results support the conclusions reported by a recent literature review indicating that there are no significant differences in survival rates in the first few years of compensated diabetics compared to a healthy comparison group [28]. Other systemic diseases taken into consideration in the current analysis did not show statistical significance, and this is in agreement with data reported by other studies that found no correlations between general health and early implant failures [11,12,13,17]. A higher early failure risk with an odds ratio of 2.26 [1.32–3.86] (confidence interval 95% of 1.32–3.86) and *p* = 0.003 was recorded in the current study for implant placement in the posterior maxillary area. Implant placement in the posterior region of the maxilla has also been reported by other studies to be a significant risk factor for early failures [6,8,24]. The lower bone density and the presence of poor bone quality could be reasons that explain why this happens. Insertion torque was correlated with primary implant stability, which in turn was correlated with early failure [29]. Since high insertion torque may cause adverse biological events by altering angiogenesis and inducing localized bone necrosis [30], the use of an insertion torque of 20–30 Ncm has been suggested [8,29,31,32]. In the present study, insertion torques of <25 Ncm and 25–35 Ncm were found not to be correlated with early implant failures, while a significant statistical correlation was found with an insertion torque of ≥35 Ncm. However, these outcomes should be interpreted cautiously because of the limited sample size of included patients. With respect to other implant-related variables taken into account, cylindrical compared to conical implant design presented a higher risk of early implant failure. This outcome could be linked to the better primary stability of conical implants compared to cylindrical ones [33]. In this regard, however, it is necessary to underline that cylindrical and hollow-cylinder implants, although frequently used in the past, are now rare and have largely disappeared from clinical practice, making it difficult to monitor them in very long-term follow-up. According to analysis carried out, tissue-level implants (non-submerged), as compared to bone-level implants (submerged), presented a higher risk of early implant failure. Our results are in agreement with data reported by a recent systematic review and meta-analysis indicating that implants placed with a non-submerged healing method could present a higher risk of early implant failure [34,35]. This could be justified by communication with the oral cavity that could involve a higher possibility of inflammatory infiltration [35,36]. An interesting data point emerged from our comparative analysis of the various non-submerged implants examined: tissue-level Laser-Lok Biohorizons implants present a lower and statistically significant early failure rate compared to other tissue-level implants [36,37]. These implants are characterized by the presence of a laser-microgrooved collar surface which has been proven to be capable of promoting the creation of a physical attack of supra-crestal connective tissue. Therefore, it could be deduced that the presence of a connective seal, created at the level of the implant collar, can reduce, in the case of non-submerged implants, the apical progression of a possible inflammatory infiltrate. However, this hypothesis still needs to be proven. Compared to other implant brands, BioHorizons Laser-Lok bone-level implants also showed a lower risk of early failure. These implants possess the same laser-microgrooved collar surface, and this could justify the results obtained. However, when evaluating the specific impact of implant collar surface characteristics, it should be recognized that implants by different brands may differ in ways other than implant collar surface characteristics (e.g., in implant surface or thread geometry). Results obtained from GEE analysis did not reveal any significant correlation between short and narrow implants and early failure. Short implants were found to be statistically associated with early implant failures in several studies [6,17,19,21], while implants with narrow diameters were found to be at a higher risk only in the study by Alsaadi et al. [8], but this data was not confirmed by other studies [7,13,19,24]. It must still be considered that short implants and/or narrow-diameter implants are often inserted in sites without adequate bone volume, which may confound the analyses. Another reported risk factor which did not correspond with results of the current study is the history of periodontitis [4,21]. However, to date there is still a controversial debate on this topic as several studies have not found a higher risk for early implant failures in periodontally susceptible individuals [9,11,22,31]. This is aligned with our results. For this possible risk factor, it must be emphasized that a “history of periodontitis” could indirectly indicate the presence of compromised sites at the time of implant insertion. Therefore, it would be the absence of adequate “true” bone volume that would predict early implant failures, rather than the susceptibility to periodontitis.

In the present study, no previous antibiotics had been administered in any of the reported incidents of implant failure. However, the occurrence of postsurgical infections, linked to poor oral hygiene due to an increase in bacterial biofilm and the subsequent infection of the area surrounding the implant, may result in bone loss that leads to implant failure not only in the long term but also in the short term [38]. Good oral hygiene practices, both prior to dental implant placement surgery and with regular maintenance afterwards, are an important factor in the success of implant treatment [39].

The limitations of the current study are related to its retrospective design, with the following factors proving to be limitations: the lack of homogeneity, since different people have been involved at different times in patient care and data entry; baseline characteristics, since the study subjects may not be representative of the whole population; and chart selection, since data was not collected for research and resultantly some charts have been excluded due to certain crucial information being missing. Moreover, it must be noted that it was difficult to control, all at once, the large number of variables taken into consideration. Another limitation may be related to the long follow-up period of the study, during which many changes were made to the designs and surfaces of dental implants. However, all the implants studied underwent minimal changes and are currently available on the market.

Future prospective studies are needed to investigate the factors associated with early implant failures.

## 5. Conclusions

The current study’s strength was its investigation of the risk factors related to early implant failures not only in a favorable patient population, but in all patients treated in a private implant clinic over 20 years, regardless of their accompanying medical or other conditions. Patient variables related to “gender”, “smoking habits,” “systemic diseases”; surgical variables related to “implant site”, “surgical technique,” and “insertion torque”, and implant variables related to “implant design” and “implant brands” have significant associations with an increased risk of overall early implant failure in private routine practice.

## Figures and Tables

**Table 1:** Patient-related variables (n = 392).

Patient-Related Variables (n = 392)	Frequency (n)	Proportion (%)
**Age**	Mean ± SD	Min-Max
	51.2 ± 15.4	18–72
≤40 years old	125	32
41–60 years old	188	48
>60 years old	79	20
**Gender**		
Female	192	48.9
Male	200	51.1
**Smoking**		
Yes	53	13.5
No	339	86.5
**Lateral Sinus Lift**		
No	372	94.9
Yes	20	5.1
**Internal Sinus Lift**		
No	380	96.9
Yes	12	3.04
**Systemic Diseases**		
No	305	77.8
Yes	87	22.2
-Type I Diabetes Mellitus	5	1.2
-Type II Diabetes Mellitus	18	4.6
-Hypertension	26	6.6
-Arrhythmia	5	1.3
-Asthma	8	2.0
-Hypercholesterolemia	12	3.1
-Hyperthyroid	1	0.2
-Hypothyroid	3	0.8
-Gastric ulcer	1	0.2
-Osteoporosis	5	1.3
-Rheumatic diseases	3	0.8
-History of radiotherapy and chemiotherapy	55	14.3

**Table 2:** Surgery-related variables (n = 930).

Surgical-Related Variables (n = 930)	Frequency (n)	Proportion (%)
Surgical phase		
One-stage	325	34.9
Two-stage	605	65.1
Bone augmentation		
No	623	67
Yes	307	33
Barrier membrane		
No	233	75.3
Yes	697	24.7
Immmediate dental implant placement		
No	819	88
Yes	111	12
Immediate mucosal grafting		
No	837	90
Yes	93	10
Insertion torque		
≥35 Ncm	106	11.4
25–35 Ncm	623	66.9
<25 Ncm	201	21.7

**Table 3:** Implant-related variables (n = 930).

Implant-Related Variables (n = 930)	Frequency (n)	Proportion (%)
Implant site		
Anterior maxilla	167	17.9
Posterior maxilla	291	31.3
Anterior mandibular	46	5
Posterior mandibular	426	45.8
Implant diameter		
Narrow (<3.75 mm)	316	33.9
Medium (3.75–4.3 mm)	530	57.9
Wide (>4.3 mm)	84	8.2
Implant length		
Short (<10 mm)	278	29.8
Medium (10–11.5 mm)	511	54.9
Long (>11.5 mm)	141	15.3
Implant design		
Conical	805	86.5
Cylindrincal	125	13.5
Implant level		
Bone level	605	65.1
Tissue level	325	34.9
Implant brand		
Straumann SLA active	135	14.5
Biohorizons Laser-Lok Tapered	306	32.9
Biohorizons Laser-Lok Tissue level	190	20.4
Biomet 3I Osseotite	84	9.1
Sweden & Martina CSR	114	12.2
Sulzer Calcitek Threadloc	101	10.9

**Table 4:** Multivariate risk factor analysis for early implant failures using GEE (n = 930).

Variables			Univariate Analysis		Multivariate Analysis	
	Failure n (%)	Success n (%)	*p*-Value	PR (CI 95%)	*p*-Value	PR (CI 95%)
**Gender**						
Female	20 (2.2)	910 (97.8)	>0.05	1	>0.05	1
Male	34 (3.7)	896 (96.3)	0.02	0.04 (0.06–0.74)	0.01	0.25 (0.08–0.7)
**Smoking**						
Yes	42 (4.6)	888 (95.4)	0.014	1.8 (1.2–2.2)	0.04	0.18 (0.02–0.08)
No	12 (1.3)	918 (98.7)	>0.05	1		
**Lateral sinus lift**						
No	28 (3.0)	902 (97.0)	>0.05	1	>0.05	1
Yes	26 (2.7)	904 (97.3)	>0.05	1.40 (0.49–3.92)	>0.05	1.98 (0.28–3.01)
**Internal sinus lift**						
No	24 (2.5)	906 (97.5)	>0.05	1	>0.05	1
Yes	30 (3.2)	900 (96.8)	>0.05	1.24 (0.81–3.14)	>0.05	1.34 (0.9–1.6)
**Systemic diseases**						
No	32 (3.5)	898 (96.5)	>0.05	1	>0.05	1
Yes	28 (3.0)	902 (97)	>0.05	0.44 (060–2.46)	>0.05	0.4 (0.15–1-16)
**Surgical phase**						
Two-stage	14 (1.5)	916 (88.5)	>0.05	1	>0.05	1
One-stage	40 (4.3)	890 (93.7)	0.003	1.11 (0.9–1.6)	0.014	0.25 (0.08–0.76)
**Bone augmentation**						
No	28 (3.0)	902 (97.0)	>0.05	1	>0.05	1
Yes	26 (2.8)	904 (97.8)	>0.05	1.60 (0.72–3.60)	>0.05	0.73 (0.48–3.6)
**Barrier membrane**						
No	29 (3.1)	901 (96.9)	>0.05	1	>0.05	1
Yes	25 (2.6)	905 (97.4)	>0.05	1.18 (0.68–2.44)	>0.05	1.42 (0.48–3.44)
**Immmediate dental implant placemant**						
No	25 (2.6)	905 (97.4)	>0.05	1	>0.05	1
Yes	29 (3.1)	901 (96.9)	>0.05	1.42 (0.78–2.14)	>0.05	1.71 (0.57–2.89)
**Immediate mucosal grafting**						
No	27 (2.6)	903 (97.4)	>0.05	1	>0.05	1
Yes	27 (2.9)	903 (97.1)	>0.05	1.35 (0.51–2.86)	>0.05	1.82 (0.91–4.16)
**Insertion torque**						
25–35 Ncm (11%) 102	6 (0.6)	924 (99.4)	>0.05	1	>0.05	1
<25 Ncm (22%) 205	12 (1.2)	918 (98.8)	>0.05	0.58 (0.20–1.69)	>0.05	0.66 (0.50–2.96)
≥35 Ncm (67%) 623	36 (3.8)	894 (96.2)	0.004	0.18 (0.06–1.57)	0.002	0.02 (0.01–0.75)
**Implant site**						
Posterior mandibular	14 (1.5)	916 (98.5)	>0.05	1	>0.05	1
Anterior maxilla	10 (1.7)	920 (98.3)	>0.05	1.19 (0.67–2.98)	>0.05	0.9 (0.55–3.02)
Posterior maxilla	25 (2.6)	905 (97.4)	0.003	2.26 (1.32–3.86)	0.001	0.04 (0.22–1.14)
Anterior mandibular	6 (0.6)	924 (99.4)	>0.05	1.70 (0.72–4.06)	>0.05	0.76 (0.12–3.44)
**Implant diameter**						
Narrow (<3.75 mm)	19 (2.0)	111 (98.0)	>0.05	1	>0.05	1
Medium (3.75–4.3 mm)	17 (1.8)	113 (98.2)	>0.05	0.97 (0.49–1.53)	>0.05	0.57 (0.31–1.34)
Wide (>4.3 mm)	18 (1.9)	112 (98.1)	>0.05	0.51 (0.21–3.22)	>0.05	0.89 (0.12–3.45)
**Implant length**						
Short (<10 mm)	14 (1.5)	116 (98.5)	>0.05	1	>0.05	1
Medium (10–11.5 mm)	20 (2.1)	110 (97.9)	>0.05	0.90 (0.50–1.56)	>0.05	0.85 (0.37–2.66)
Long (>11.5 mm)	20 (2.1)	110 (97.9)	>0.05	0,.53 (0.31–3.34)	>0.05	0,.94 (0.22–2.99)
**Implant design**						
Conical	14 (1.5)	916 (88.5)	>0.05	1	>0.05	1
Cylindrincal	40 (4.3)	890 (95.7)	0.004	0.99 (0.07–1.49)	0.002	0.07 (0.02–0.79)
**Implant level**						
Bone level	18 (1.9)	912 (98.1)	>0.05	1	>0.05	1
Tissue level	36 (3.9)	894 (96.1)	0.001	1.92 (1.12–3.86)	0.001	0.02 (0.11–0.95)
**Implant brand**			,			
Biohorizons Laser-Lok Tissue Level	0 (0.0)	930 (99.1)	>0.05	1	>0.05	1
Biohorizons Laser-Lok Tapered	8 (0.8)	922 (99.2)	>0.05	0.15 (0.02–1.20)	>0.05	0.42 (0.18–2.34)
Straumann SLA Active	12 (1.3)	918 (98.7)	0.001	1.32 (0.17–1.57)	0.003	0.03 (0.01–0.55)
Biomet 3I Osseotite	10 (1.1)	920 (98.9)	>0.05	0.58 (0.20–1.69)	>0.05	0.17 (0.02–1.28)
Sweden & Martina CSR	11 (1.2)	919 (98.8)	0.001	0.19 (0.07–0.49)	0.002	0.09 (0.07–0.66)
Sulzer Calcitek Threadloc	14 (1.5)	916 (98.5)	0.004	0.18 (0.06–0.57)	0.004	0.08 (0.09–0.77)

## Data Availability

The data that were analyzed to reach the conclusion of this study can be requested from Renzo Guarnieri.
